# Retinal Imaging and Optical Coherence Tomography of Choroidal Haemangioma Mimicking Central Serous Chorioretinopathy

**DOI:** 10.7759/cureus.35353

**Published:** 2023-02-23

**Authors:** Davina Jugnarain, Raawiyah Mohamudally, Nedjema Chaabane-Rajah, Zia Carrim

**Affiliations:** 1 General Medicine, North Middlesex University Hospital, London, GBR; 2 Ophthalmology, The Eye Clinic, Port Louis, MUS

**Keywords:** oct (optical coherence tomography), ophthalmic oncology, retinal diseases, ocular oncology & medical retina, choroidal hemangioma, central serous chorioretinopathy

## Abstract

Intraocular tumours, such as choroidal haemangioma, can cause exudative retinal detachments, which mimic central serous chorioretinopathy. Key symptoms of a choroidal haemangioma include reduced visual acuity, visual field defects, and metamorphopsia. More rarely, it can cause photopsia, myodesopsia, and pain. Important differentials include choroidal melanoma and metastases, for which an ocular oncologist should be consulted. Prompt treatment is required for the regression of tumour and to prevent choroidal atrophy and permanent visual loss. Here, we report the case of a 44-year-old lady who was found to have a choroidal haemangioma with macular subretinal fluid, highlighting the differentiating features from other intraocular masses.

## Introduction

Haemangiomas are a class of benign tumours affecting vascular endothelium in various parts of the body. In ophthalmology, circumscribed choroidal haemangiomas can present between the fourth and sixth decades of life [1]. They can be accompanied by a serous neurosensory detachment of macula, which can mimic central serous chorioretinopathy and cause degenerative changes to the retinal pigment epithelium [1,2]. Therefore, the most common presenting symptoms are visual acuity loss (81%), visual field defects (7%), and metamorphopsia (3%) [3]. Less common symptoms include photopsia, myodesopsia, and pain [4]. The lattermost symptom is most likely a result of total retinal detachment with neovascular glaucoma [4]. A study by Shields et al. found that 53% of 200 cases with choroidal haemangioma had a visual acuity of 1.00 logMAR (20/200 Snellen) or worse on initial presentation [4]. Therefore, recognition of choroidal haemangioma and its treatment is important to maintain visual potential. The mainstay of treatment for choroidal haemangioma is typically verteporfin photodynamic therapy, which improves or maintains vision, by promoting tumour regression and reabsorption of subretinal fluid [5]. Shields et al. also found that misdiagnoses were made in 71% of 200 cases with choroidal haemangioma [4].

In this case report, we present high-quality images from a case of a 44-year-old female patient with a circumscribed choroidal haemangioma and serous macular detachment, thus initially mimicking central serous chorioretinopathy.

## Case presentation

**Figure 1:**
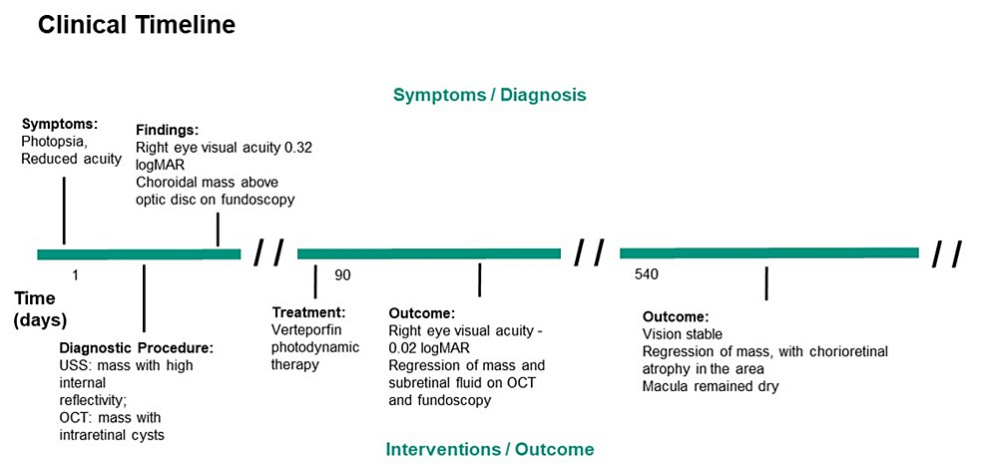
Clinical timeline. Timeline of events where a 44-year-old South Asian female was diagnosed and treated for choroidal haemangioma. USS: ultrasonography; OCT: optical coherence tomography.

A 44-year-old lady presented with a six-month history of photopsia and reduced vision in her right eye. Her past medical history was notable for systemic hypertension alone, but she took no regular medications. She had already had an ophthalmological evaluation elsewhere and was told that she may have central serous chorioretinopathy. The clinical timeline is summarised in Figure 1.

**Figure 2:**
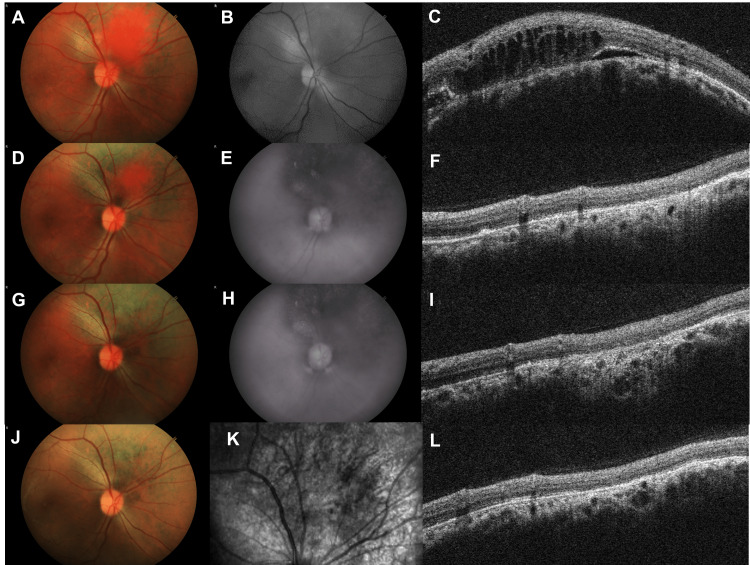
Fundoscopy and OCT. Fundus examination revealed a circumscribed choroidal haemangioma superior to the optic nerve, visible as a round mass with a dark red glow, with no atrophic retinal changes (A). It was better visualised with red-free fundus photography illustrating a shadow superior to the optic nerve (B). OCT demonstrated a raised mass with subretinal fluid (C). Fundus photography showed a reduction in the mass four months later post-photodynamic therapy (D). Atrophic changes and pigmentary disturbance remained around the mass (E). OCT imaging showed a reduction in the size of the mass and reduced subretinal fluid (F). One year later, the resolution of the mass was seen with atrophic changes in the previous area (G, H). OCT demonstrated resolution of the mass and subretinal fluid (I). At two years of follow-up, the mass remained in remission (J) with an atrophic area in the previous location of the mass on OCT (K). OCT showed no further change or changes to the thickness parameters of the retina (L). OCT: optical coherence tomography.

**Figure 3:**
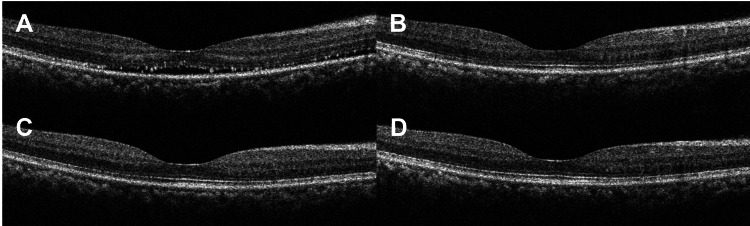
Subretinal fluid on OCT. Shallow subretinal fluid on OCT upon initial presentation (A) had resolved four months later post-photodynamic therapy (B). This remained stable at one year (C) and two years (D) of follow-up. OCT: optical coherence tomography.

Examination on the initial presentation found her visual acuity to be 0.32 logMAR (20/42 Snellen) in the right eye and -0.10 logMAR (20/16 Snellen) in the left. Both eyes were quiet and had clear media as well as normal intraocular pressures and optic disc appearances. While the retina in the left eye was normal, there was shallow subretinal fluid in the right macula. This seemed to be tracking down from a large choroidal mass with a dark red glow, situated just above the optic disc (Figure 2A and B). Ultrasonography (USS) of the mass revealed high internal reflectivity and acoustic brightness. The thickness was measured at 4 mm at its maximum point of elevation. Optical coherence tomography (OCT) revealed intraretinal cysts in association with the mass (Figure 2C). The presence of subretinal fluid in the macula was also confirmed (Figure 3A). A circumscribed choroidal haemangioma, a benign hamartoma, was suspected based on clinical findings and imaging features. There were no clinical features of Sturge-Weber syndrome. Despite reasonable certainty about the diagnosis, it was felt that a choroidal melanoma needed to be excluded, and a referral was made to ocular oncology.

Four months later, she returned for review having had confirmation of our diagnosis and having undergone verteporfin photodynamic therapy. Her visual acuity had improved to -0.02 logMAR (20/19 Snellen) in the eye of concern, whereas the left was stable. Examination of the retina showed an atrophic area at the previous site of the haemangioma, with mild pigmentary disturbance and minor residual elevation (Figure 2D and E). OCT showed the resolution of subretinal fluid (Figures 2F and 3B). She reported an improvement in her vision but felt her colour perception was still impaired.

Over an additional 18-month period of follow-up, her vision was noted to be objectively stable. Biomicroscopically, progressive chorioretinal atrophy was observed in the area of the retina where the haemangioma had been present. The macula remained dry on OCT (Figure 3).

## Discussion

We have outlined a case of choroidal haemangioma with associated macular subretinal fluid, initially mistaken for central serous chorioretinopathy, a common mimic. In our case, this responded well to verteporfin photodynamic therapy and regressed over the course of two years, leaving an area of chorioretinal atrophy. To reduce the extent of atrophy and preserve visual potential, correct diagnosis and prompt treatment are necessary. We will outline the differential diagnoses for a choroidal haemangioma and the investigations that can be used to discern them.

Differential diagnoses for choroidal haemangiomas include central serous chorioretinopathy, choroidal melanomas, and metastases [3]. On fundus examination, choroidal haemangiomas have distinguishing characteristics from these other differentials. For example, choroidal haemangiomas are round or oval masses with an orange colour similar to normal choroid and, when circumscribed, may be demarcated by a brown halo related to compression of the normal choroidal vasculature [6]. They are typically situated within two-disc diameters of the optic nerve or foveola or both. The overlying retina undergoes degenerative changes such as atrophy, calcification, and fibrous metaplasia. The latter may give the choroidal haemangioma a brown appearance [7]. This makes choroidal haemangiomas harder to distinguish from choroidal melanomas, which are usually brown or grey neoplasms with a thickness of at least 2 mm, and irregular, poorly demarcated borders [3,8]. Choroidal metastases can have a similar orange colour to choroidal haemangiomas, particularly for primary malignancies of renal, thyroid, or carcinoid origin [3]. Like choroidal haemangiomas, central serous chorioretinopathy typically presents in middle age. Although hyperpermeability of the choroid underlies the development of central serous chorioretinopathy, choroidal thickness will typically diffusely be 500 μm, whereas thickening may be 1000-2000 μm in a discrete manner for choroidal haemangiomas [6]. There are several investigations that can differentiate a choroidal haemangioma from other choroidal masses and central serous chorioretinopathy.

Investigations that may better characterise choroidal haemangioma include enhanced-depth imaging OCT, USS, fluorescein angiography, and indocyanine green angiography. As found in this report, OCT can show subretinal fluid and cystic changes overlying the elevated mass [9]. USS can show a fusiform lesion, which typically has high internal reflectivity [9]. Fluorescein angiography typically shows hyperfluorescence of large choroidal vessels in the early stage, with prolonged staining of the lesion and subretinal fluid in the late stage [9,10]. Whereas fluorescein angiography can show focal retinal pigment epithelium leaks that evolve into a mushroom pattern, indocyanine green angiography typically shows filling and hypercyanescence of the vascular lesion, where washout of the central portion occurs in the late phase [9,10]. These findings can distinguish choroidal haemangioma from other intraocular tumours.

## Conclusions

Despite delayed diagnosis, this patient experienced good resolution of subretinal fluid and regression of the choroidal haemangioma after photodynamic therapy. However, delayed recognition and, thus, treatment of a choroidal haemangioma are associated with poor long-term vision. Therefore, intraocular tumours presenting with retinal symptoms should warrant careful examination of the posterior fundus and ancillary investigations, such as OCT, USS, and fluorescein or indocyanine green angiography, to establish the correct diagnosis promptly.
